# EHMTI-0302. SUNCT syndrome, case report

**DOI:** 10.1186/1129-2377-15-S1-C33

**Published:** 2014-09-18

**Authors:** A Kuqo, J Kruja

**Affiliations:** 1 Neurology, UHC Mother Teresa, Tirana, Albania; 2 Neurology, University of Medicine, Tirana, Albania

## Introduction

The short-lasting primary headache syndromes may be conveniently divided into those exhibiting marked autonomic activation and those without autonomic activation. The former group comprises chronic and episodic paroxysmal hemicrania, short-lasting unilateral neuralgiform headache with conjunctival injection and tearing (SUNCT syndrome) and cluster headache. SUNCT is relatively rare, with a recent study showing a prevalence of 6.6/100,000 and an incidence of 1.2/100,000. The disorder has a male preponderance, with a sex ratio of 2:1.The typical age of onset is between 40 and 70 years, with a mean age of onset at 48 years.

## Methods

We report the case of a 73 years old man, with a history of two months of right side retro-orbital severe stabbing headache attacks that last 70 seconds with a frequency of 5 to 7 attacks per hour. During the attacks he had ipsilateral conjuctival injection, periorbital and facial redness and sweating, eyelid edema, ptosis tearing and nasal congestion (video).

The patient was treated first with Gabapentin 600 mg/d but it was shown ineffective. We start the treatment with sodium valproate up to 1500 mg/d and after one month the patient had a significant reduction of attacks and their severity.

## Conclusion

The most of drugs used in treatment of primary headaches are ineffective in the treatment of SUNCT syndrome. Sodium valproate is B recommendation of AAN, and may be good choice in treatment of this syndrome.

No conflict of interest.

